# Lipid Parameters and Proprotein Convertase Subtilisin/Kexin Type 9 in Healthy Lebanese Adults

**DOI:** 10.3390/metabo12080690

**Published:** 2022-07-26

**Authors:** Marie-Hélène Gannagé-Yared, Elie Naous, Anis Al Achkar, Wadih Issa, Ghassan Sleilaty, Vanda Barakett-Hamade, Marianne Abifadel

**Affiliations:** 1 Department of Endocrinology, Faculty of Medicine, Saint-Joseph University, Beirut 11-5076, Lebanon; elie-naous@hotmail.com (E.N.); wadihissa97@gmail.com (W.I.); 2 Laboratory of Hormonology, Hôtel-Dieu de France Hospital, Department of Laboratory Medicine, Faculty of Medicine, Saint-Joseph University, Beirut 11-5076, Lebanon; anis_achkar94@hotmail.com; 3 Department of Biostatistics and Clinical Research Center, Faculty of Medicine, Saint-Joseph University, Beirut 11-5076, Lebanon; ghassan.slailaty1@usj.edu.lb; 4 Laboratory of Biochemistry, Hôtel-Dieu de France Hospital, Department of Laboratory Medicine, Faculty of Medicine, Saint-Joseph University, Beirut 11-5076, Lebanon; vanda.barakett@hdf.usj.edu.lb; 5 Laboratory of Biochemistry and Molecular Therapeutics (LBTM), Faculty of Pharmacy, Pole Technologie-Santé (PTS), Saint-Joseph University, Beirut 11-5076, Lebanon; marianne.abifadel@usj.edu.lb

**Keywords:** HDL-C, non-HDL-C, triglycerides, lipoprotein(a), PCSK9

## Abstract

Background: High levels of non-HDL cholesterol (non-HDL-C), triglycerides (TG), lipoprotein (a) (Lp(a)), and Proprotein convertase subtilisin/kexin type 9 (PCSK9) as well as low levels of HDL-C are strongly associated with cardiovascular disease (CVD). Our study aims to estimate the prevalence of dyslipidemia and high Lp(a) in the Lebanese population and to study the relationship of these variables with gender, age, body mass index (BMI), and PCSK9. Methods: This cross-sectional study was carried out on a sample of healthy volunteers aged 18 to 65. Blood samples were drawn from volunteers for total cholesterol (TC), HDL-C, TG, PCSK9, and Lp(a) measurements. Non-HDL-C was calculated by subtracting HDL-C from TC. Results: In total, 303 volunteer subjects with an average age of 38.9 years were included in the study. Respectively, 44%, 29.8%, and 44% of men had high non-HDL-C and TG with low HDL-C versus 23.5%, 8%, and 37% in women. Non-HDL-C and TG were significantly higher in men than in women, while the reverse was observed for HDL-C (*p* < 0.0001 for the three comparisons). Non-HDL-C and TG were significantly correlated with age and BMI (*p*< 0.0001 for all correlations), while HDL-C was inversely correlated with BMI (*p* < 0.0001) but not with age. Abnormal Lp(a) levels (≥75 nmol/L) were found in 19.1% of the population, predominantly in women (24.1% versus 13.4% in men, *p* = 0.004). The median PCSK9 and its interquartile was 300 (254–382) ng/L with no gender difference (*p* = 0.18). None of the following factors: gender, age, BMI, non-HDL-C, HDL-C, or TG, were independently associated with Lp(a), while PCSK9 was significantly correlated with age, non-HDL-C, and TG in both men and women and inversely correlated with HDL-C in men. Dyslipidemia is very common in the Lebanese population and is associated with age, high BMI, and male sex. Lp(a) is higher in women without any correlation with the lipid profile, whereas PCSK9 is associated with non-HDL-C and TG. Further studies are needed to evaluate the potential role of Lp(a) and PCSK9 in predicting CVD in healthy populations.

## 1. Introduction

The lipid profile and its components (total cholesterol (TC), low-density lipoprotein-Cholesterol (LDL-C), high-density lipoprotein-Cholesterol (HDL-C), and triglycerides (TG)) are among the most commonly ordered laboratory tests in clinical practice. High levels of LDL-C and TG and low levels of HDL-C are associated with an increased risk of atherosclerotic cardiovascular disease (ASCVD) [1]. 

Lipoprotein (a) (Lp(a)) is another lipoprotein associated with increased ASCVD [2]. Its composition resembles that of LDL-C and it is characterized by the presence of a covalent bond between apolipoprotein A and apolipoprotein B100 [3]. Although the exact function of Lp(a) has not yet been determined, numerous studies have shown that it is an independent risk for ASCVD, particularly in subjects with concomitant elevation of LDL-C [4]. Lp(a) is primarily genetically regulated [5], with higher levels in Blacks compared to Whites [5]. Its relationship with gender, age, and Body Mass Index (BMI) showed contradictory results, with some studies showing increased levels with age [6,7], in women [8,9], or in subjects with lower BMI [8]. In the Framingham offspring study, Lp(a) has been shown to be inversely associated with TG but not with TC and LDL-C [10], while other studies showed a positive correlation with LDL-C but not HDL-C [11] or no association with the lipid profile [12]

Proprotein convertase subtilisin/kexin type 9 (PCSK9) is the ninth member of the proprotein convertase family [13]. In 2003, Abifadel et al. [14] described its link with cholesterol metabolism and familial hypercholesterolemia. It is a serine protease produced primarily by the liver, where it mediates LDL receptor (LDLR) degradation in lysosomes, resulting in fewer LDLRs on the cell membrane [15]. Therefore, PCSK9 decreases LDL-C clearance from circulation and elevates plasma LDL-C [16]. Elevated levels of circulating PCSK9 have also been associated with increased LDL-C, TG [16] and worse cardiovascular outcomes [17]. PCSK9 levels varies according to gender, age, and BMI, with higher levels in women compared to men [16,18], postmenopausal vs. premenopausal women [16,18,19], and in subjects with a higher BMI [20,21]. 

Dyslipidemia is very prevalent worldwide. In the United States (US), this prevalence is as high as 75 to 85% in patients with premature coronary heart disease (CHD) compared with 40 to 48% in age-matched controls without CHD [22]. The Middle East is also a part of the world where dyslipidemia is highly prevalent [23]. In Lebanon, few cross-sectional studies looked at this prevalence in adult [24,25] or pediatric populations [26,27]. A single study published in 1999 on a population of a tertiary hospital staff found that the respective prevalence of high LDL-C levels and Lp(a) were 27% and 20% [24]. In Lebanese children, the prevalence of high non-HDL-C and TG are, respectively, 9.2% and 26.6% [26], and the prevalence of high Lp(a) is 14.4% [27]. No other studies have evaluated the lipid profile (TC, HDL-C, TG, Lp(a), and PCSK9) in the Lebanese adult population and its relation to gender, age, and BMI. 

The purpose of this study is to determine the prevalence of abnormal components of the lipid profile and to study, for the first time, the relationship between the classic lipid profile (non-HDL-C, TG, HDL-C) and both Lp(a) and PCSK-9 in a sample of the Lebanese adult population. We will also look at the variation in Lp(a) and PCSK-9 according to gender, age, and BMI. 

## 2. Methods

### 2.1. Population

This is a cross-sectional study conducted on a sample of healthy volunteers aged between 18 and 65 years. A questionnaire was distributed to healthy hospital visitors and employees inviting them to participate in the study. Thus, a convenience sampling was opted for. The authors’ institution, Hôtel-Dieu de France hospital, is a tertiary care facility treating patients from the whole country; therfore, the included subjects came from a mix of rural and urban settings. Blood samples were collected at our institution laboratory between October 2020 and January 2021 among the consenting participants. 

Data were recorded through both an interview and a questionnaire. Exclusion criteria were the following: age < 18 years old or >65 years old; pregnancy or childbirth less than a year ago or breastfeeding; chronic diseases such as chronic renal failure, chronic heart failure, chronic liver failure, thyroid dysfunction (abnormal TSH), diabetes, cancer, autoimmune or inflammatory diseases, or psychiatric diseases. Subjects taking a lipid lowering drug or drug that can affect lipid profile such as beta blockers, glucocorticoids, salicylates, isotretinoin, atypical antipsychotics, and the contraceptive pill were also excluded from the study. Current hospitalization, hospitalization during the last month, or excessive alcohol consumption (>7 drinks per week) were also exclusion criteria.

On the day of collection, participants’ weight and height were collected. BMI was calculated using the formula weight/(height)^2^, with weight expressed in kilograms and height in meters. The weight was taken on the same scale, with light clothing, and the height was measured without shoes. BMI was divided into four subclasses: underweight for BMI < 18 kg/m^2^, normal weight for BMI between 18 and 25 kg/m^2^, overweight for BMI between 25 and 30 kg/m^2^, and obese for a BMI greater than 30 kg/m^2^. 

An informed consent was signed by each participant. The study had the approval of the ethics committee of Hôtel-Dieu de France hospital (CEHDF1760).

### 2.2. Biological Parameters

Peripheral blood was collected in the morning between 8 and 12 am after a 12-h fasting period. Samples were analyzed the same day for TC, HDL-C, TG, Lp(a), and TSH. Part of the samples were stored at −80 °C for later PCSK9 measurements. 

### 2.3. Lipid Profile

TC, HDL-C, and TG measurements were performed on a Vitros 5.1 FS machine (Ortho-Clinical Dignostics, Inc. Raritan, NJ, USA). Non-HDL-C was calculated by subtracting HDL-C from the TC. LDL-C was calculated using the Friedewald formula where LDL-C, TC, and HDL-C are expressed in mmol/L (LDLC = TC − (HDL-C + TG/2.2)). Based on the lipid thresholds established by the National Cholesterol Education Program (NCEP guidelines) [28], lipid values were defined as follows: (1) for non-HDL-C—normal (≤3.3 mmol/L), borderline (3.3–4.1 mmol/L), high (>4.1 mmol/L), (2) for TG—normal (≤1.69 mmol/L, borderline (1.69–2.25 mmol/L), high (2.25–5.65), very high (>5.65 mmoL/L), (3) for HDL-C—low (<1 mmol/L in men and <1.3 mmoL/L in women), normal (≥1 mmol/L in men and ≥1.3 mmoL in women).

### 2.4. Lp(a) Measurement 

The assay is a particle-enhanced immunoturbidimetric assay (Cobas Integra 400 plus system, Roche Diagnostics, Basle, Switzerland). In this method, human Lp(a) agglutinates with latex particles coated with anti-Lp(a) antibodies. The precipitate is determined by turbidimetry at 659 nm. The measurement range varies from 7 to 240 nmol/L with a detection limit of 7 nmol/L. Samples greater than 240 nmol/L were diluted. According to the manufacturer, this is the threshold value which indicates an increased risk is 75 nmol/L. The conversion factor from nmol/L to mg/dL is (nmol/L + 3.83) × 0.4587 = mg/dL. The intra and inter-coefficient of variation is less than 4%.

### 2.5. PCSK9 Measurement 

PCSK9 concentrations were measured by an enzyme-linked immunosorbent assay (Human PCSK9 ELISA Kit, R&D Systems, Cambridge, UK) using a spectrophotometric method at 450 nm. This assay employs a quantitative sandwich enzyme immunoassay technique using a monoclonal antibody specific for human PCSK9 that has been pre-coated onto a microplate. The PCSK9 from standards and samples are bound by the immobilized antibody and a second polyclonal antibody specific for human PCSK9 is then added to the wells. The CV of the reaction is less than 7%. According to the manufacturer, the normal range established in healthy volunteers is from 177 to 460 ng/mL.

### 2.6. TSH Measurement

TSH was measured by a chemiluminescent assay on the Immulite 2000 automate (Siemens, Washington, DC, USA). The sensitivity of the assay is 0.004 IU/L and the CV of the reaction is less than 6% for values within the normal range.

### 2.7. Statistical Analysis

Quantitative variables were tested for normality distribution using the Kolmogorov–Smirnov test. In case the variable departed significantly from normality, it was expressed as median and interquartile range (IQR) (quartile 1–quartile 3). Categorical and normally distributed continuous variables were expressed as frequencies and mean ± standard deviation, respectively. 

Original Lp(a) values reported semi-quantitatively as Lp(a) values < 7 nmol/L were set to 6 nmol/L and were analyzed using non-parametric methods. Chi-square test, independent t test, and Mann–Whitney U test were used in univariate analyses. Spearman’s correlation was used to assess the association between quantitative variables, and its 95% confidence interval (95%CI) was built using the Caruso and Cliff method. A multiple linear regression model was built with natural logarithm (Ln) of Lp(a) as continuous dependent variable and age, Ln of BMI, and Ln of lipid parameters as independent factors. The model’s R2 and Cook’s influence statistics were calculated. The statistical analysis was performed using SPSS (IBM Corp. Released 2019, SPSS Statistics for Windows Version 26.0, Armonk, NY, USA).

## 3. Results

**Anthropometric and socioeconomic characteristics of the population (Table 1)**. A total of 303 subjects were included in the study (46.5% men and 53.5% women). The mean age was 38.9 ± 12.5 years, with no significant difference between genders (*p* = 0.94). The mean BMI was significantly higher in men compared to women (respectively 27.90 ±5.11 vs. 24.42 ± 4.59; *p* <0.001). In the entire sample, 20.8% were obese and 31.7% overweight (Table 1). Finally, 29 subjects (9.6%) were hypertensive.

**Non-HDL-C, HDL-C, and TG values in the entire sample and in relation with gender, age, and BMI**. Results of non-HDL-C, TG, and HDL-C in the entire sample and by gender are shown in Table 2. Respectively, 28.7% and 33.0% have borderline and high non-HDL-C. In addition, 27.7% of the subjects have borderline or high TG, and 1.7% very high levels. Finally, 44.0% of men and 37.0% of women have low-HDL-C. A significant difference is observed between men and women for TG, HDL-C, and non-HDL-C (*p* < 0.0001 for all three comparisons). Non-HDL-C and TG were significantly correlated with age and BMI (Spearman’s rho respectively 0.39 (95%CI 0.29–0.49) and 0.30 (95%CI 0.19–0.40) for non-HDL-C and 0.33 (95%CI 0.22–0.43) and 0.39 (95%CI 0.28–0.48) for TG, all *p*-values < 0.0001). Finally, HDL-C was inversely correlated with BMI (Spearman’s rho −0.40 (95%CI −0.49–−0.30), *p* < 0.0001) but not with age (Spearman’s rho −0.05 (95%CI −0.16–0.07), *p* = 0.402). (Table 3).

**Lp(a) values in the entire sample and in relation with gender, age, BMI, and other lipid parameters**. Abnormal Lp(a) levels (≥75 nmol/L) were observed in 19.1% of the whole sample (13.5% in men and 24.1% in women (*p*-value = 0.044)). The proportion of subjects with a Lp(a) level ≤ 7 nmol/L and between 7 and 75 nmol/L was, respectively, 23.1% and 57.8% (Table 2). Only 7.6% of the subjects had values above 120 nmol/L.

Lp(a) was not correlated with age (Spearman’s rho 0.09 (95%CI −0.02–0.20, *p* = 0.112)), nor with BMI (Spearman’s rho 0.03 (95%CI −0.08–0.15, *p* = 0.553)), even when the analysis was performed separately for men and women (Table 3).

Lp(a) was significantly correlated with HDL-C (rho 0.13 (95%CI 0.02–0.24, *p* = 0.025) but not with TG nor non-HDL-C (rho −0.06 (95%CI −0.17–0.05, *p* = 0.277) and 0.04 (95%CI −0.07–0.15, *p* = 0.489), respectively). Overall, the correlation between Lp(a) and PCSK9 was weak (rho 0.11 (95%CI −0.01–0.22, *p* = 0.062)), but was higher and significant in women (rho 0.22 (95%CI 0.06–0.36, *p* = 0.005)) (Table 3).

**PCSK9 values in the entire sample and in relation with gender, age, BMI, and other lipid parameters**. 

The median PCSK9 and its interquartile was 300 (254–382) ng/mL, with no gender difference (*p* = 0.18). The 2.5th and 97.5th PCSK9 percentiles were, respectively, 146 and 687 ng/mL. PCSK9 correlated significantly with age (rho 0.22 (95%CI 0.11–0.33, *p* < 0.0001), mostly in women (rho 0.32 (95%CI 0.17–0.45, *p* < 0.0001)) but not in men (rho 0.13 (95%CI −0.04–0.29, *p* = 0.140)) (Table 3). 

Overall, PCSK9 also correlated with non-HDL-C (rho 0.22 (95%CI 0.11–0.33, *p* < 0.0001)) and TG (rho 0.21 (95%CI 0.10–0.32, *p* < 0.0001)). This pattern was found in men, respectively, for non-HDL-C and TG (rho 0.35 (95%CI 0.19–0.49, *p* < 0.0001, and 0.33 (95%CI 0.17–0.48, *p* < 0.0001)) and in women (respectively, 0.17 (95%CI 0.02–0.32), *p* = 0.03, and 0.21 (95%CI 0.06–0.36), *p* = 0.007). In addition, in men, PCSK9 was inversely correlated with HDL-C (rho-0.20 (95%CI −0.34–−0.03, *p* = 0.017)) (Table 3).

**Multiple linear regression analysis with PCSK9 and Lp(a) as the dependent variables.**

A multiple linear regression analysis with LnLp(a) as a dependent variable and gender, age, BMI, non-HDL-C, TG, HDL-C, and PCSK-9 as independent variables was performed (Table 4). None of the included independent variables was independently associated with Lp(a). A similar regression with LnPCSK9 as a dependent variable was performed. Ln TG and age were associated with Ln PCSK9 (*p* = 0.002 and *p* = 0.042, respectively) (Table 5). A separate regression for LnPCSK9 performed in men and women showed that age and Ln(lp(a) were independently associated with PCSK9 in women (respective *p*-values 0.011 and 0.007), while in men, none of the independent variables achieved significance.

## 4. Discussion

In this study conducted on 303 volunteer subjects, we measured the lipid profile, Lp(a), and PCSK9 values. Respectively, 44%, 29.8%, and 44% of men had high non-HDL-C, high TG, and low HDL-C versus 23.5%, 8%, and 37% for women. Non-HDL-C and TG were significantly correlated with age and BMI while HDL-C was inversely correlated with BMI, but not with age. Non-HDL-C is not optimal (>3.3 mmol/L) in 67% and is high (>4.1 mmol/L) in 33% of our population. Worldwide, according to the World Health Organization Global Health Observatory, in 2008, abnormal total cholesterol (>4.9 mmol/L) is the highest in Europe (54% for both sexes), followed by North and South America (48% for both sexes), whereas Africa and Southeast Asia had the lowest prevalence (22.6% and 29.0%, respectively) [29]. This suggests that in our population, the prevalence of high cholesterol levels is between that of Western countries and Africa. Of note, the prevalence of high non-HDL-C has increased since 1999, at which date high LDL-C was observed in 27% of the population [24]. Presumably, this could be a consequence of unfavorable changes in dietary habits and lifestyle behaviors, which were exacerbated during the COVID-19 lockdown [30]. This trend is corroborated by data from Jordan, where the prevalence of hypercholesterolemia and hypertriglyceridemia has increased from 23% to 44.3% and from 23.8% to 41.9% between 1994 and 2017 [31]. The latter observations raise the assumption of a large increase in both plasma cholesterol and TG levels over time in low and middle-income countries in comparison to high-income countries, which witnessed no to little change in plasma TC and non-HDL-C [29]. Low HDL-C values were the most prevalent form of dyslipidemia in our sample. This is consistent with what is observed in the European population, where the prevalence of low HDL-C is 33% in males and 40% in women [32], and in Africa, where this prevalence is 37.4% [33]. 

We also found that BMI is positively correlated with TG and non-HDL-C and inversely correlated with HDL-C, confirming that overweight and obesity are risk factors for dyslipidemia. This finding has also been observed in other Middle Eastern studies [31,34,35]. In the Jordanian study, low HDL-C and high LDL-C were independently associated with obesity and waist circumference [31]. Similar to our results, in the United Arab Emirates study, age and male sex were predictive factors for dyslipidemia [35]. The android fat distribution could explain the greater prevalence of dyslipidemia in male sex. Moreover, 68.1% of our male participants were overweight or obese; this might explain the high prevalence of dyslipidemia in our sample.

A fifth of our population had an elevated Lp(a) level (≥75 nmol/L). Lp(a) was higher in females and was not found to be independently associated with gender, age, BMI, non-HDL-C, TG, or HDL-C. Lp(a) is a well-known cardiovascular risk factor. According to the National Cholesterol Education Program (NCEP), the threshold considered as a cardiovascular risk factor has been established at 30 mg/dL, which is equivalent to 75 nmol/L [28]. However, the European Atherosclerosis Society stated that, in subjects with an intermediate or high cardiovascular risk, the risk is significantly increased when Lp(a) is above 120 nmol/L or 50 mg/dL [36]. A recent study by the National Heart, Lung, and Blood Institute (NHLBI) Working Group estimated that the overall prevalence of elevated Lp(a) (greater than or 50 mg/dL) is 30% in Africa, 25% in South Asia, 20% in North America, Europe, and Oceania, 15% in Latin America, and 10% in Asia [37]. In addition, in a large study conducted in the US, 35% of the population had a Lp(a) level greater than 30 mg/dl [38], with higher values in women than in men. This prevalence is lower compared to that found in North America, Europe, and Oceania, since the threshold used in our study is 75 nmol/L instead of 120 nmol/L. The lower prevalence in our population may suggest that the high cardiovascular risk in the Lebanese population is more related to an abnormal lipid profile (non-HDL-C and high TG, low HDL-C) rather than to high levels of Lp(a). Another study from North Korea found lower Lp(a) values (average of 19.6 nmol/L) [39], suggesting that the prevalence of high Lp(a) in the Lebanese population is between that of the North American and South Asiatic populations. Of note, the prevalence of high Lp(a) in our sample did not change in comparison to the previous Lebanese report (19.1% vs. 20.0%), confirming the genetic determination of this lipoprotein, which is not the case for non-HDL-C. The issue of to what extent gender, age, and BMI influences Lp(a) levels remains unresolved. The gender difference we observed was also found in the Framingham offspring study [9] as well as in the Japanese population [8] and in Arab adolescents [40]. Similar to our results, several studies did not find any association between Lp(a) and age [12,41]. On the contrary, the Sweden Monica study found a weak but significant relation between Lp(a) and age in both sexes [7], and in the Kaiser Permanente Women Twins Study, a weak positive association was only found among Blacks [6]. Finally, we did not found any relationship between Lp(a) and BMI, similar to several other studies [10,40,41], while, on the other hand, a Japanese study [8] demonstrated lower Lp(a) levels in obese subjects. Finally, we found that Lp(a) is significantly correlated with HDL-C but not with non-HDL-C and TG, despite the biochemical and structural similarity with LDL-C [5]. A study conducted by Bovet et al. demonstrated a positive correlation between Lp(a) and LDL-C levels but not with HDL-C [11]. However, the Framingham study demonstrated that Lp(a) is negatively correlated with TG [10], suggesting contradictory results for the association between Lp(a) and the other lipid components that could be explained by ethnic or genetic differences between countries. 

Finally, no significant gender difference was found for PCSK9 levels. Contrary to our results, other studies have shown that PCSK9 levels were higher in women in comparison to men [16,18,19]. We also found that the PCSK9 was correlated with age only in women. Lakoski et al. [16] showed that women have higher levels of PCSK9 than men and that postmenopausal women had significantly higher plasma levels of PCSK9 than did premenopausal women, while there was no significant relationship between PCSK9 levels and age in men. We also did not find any relationship between PCSK9 and BMI. Higher BMI score was associated with higher levels of PCSK9 in a Sub-Saharan African Population of Patients with Obesity [20], a finding that was not also observed in the current study. On multivariate analysis, high levels of PCSK9 were significantly correlated with TG, a result similar to that of Lakoski et al. [16]. 

In terms of limitations, our sample was relatively small to be able to predict accurate prevalence of an abnormal lipid profile in the Lebanese population. Second, results related to PCSK9 should be analyzed with caution. The lack of standardized, and the new availability of commercial, PCSK9 assays may explain differences across studies. Further standardization of PCSK9 kits should be performed to establish normal values in healthy subjects and to determine the threshold which indicates an increased risk of ASCVD. However, it is worthy to mention that our study is the first one to re-assess the lipid profile in a sample of adult healthy Lebanese subjects since 1999 and to study the PCSK9 relationship with the other lipid parameters in the same population. 

## 5. Conclusions

The current study documents the high prevalence of dyslipidemia in the Lebanese population, which has increased in the last decades. Age and BMI are the major factors associated with abnormal lipid profile. Lp(a) was higher in women and not correlated with age, BMI, nor lipid profile. PCSK9 was only correlated with age, non-HDL-C, and TG in both men and women and inversely correlated with HDL-C in men. Since PCSK9 and Lp(a) levels are two predictors of coronary artery calcification in asymptomatic patients with familial hypercholesterolemia, future studies are needed to further elucidate the role of Lp(a) and PCSK9 in cardiovascular disease in Middle Eastern populations.

## Figures and Tables

**Table 1:** Baseline demographic, anthropometric, and lipid characteristics of the total population, with men and women taken separately.

	Total Population (n = 303)	Men (n = 141)	Women (n = 162)	*p*-Value
**Age** years	38.9 ± 12.5	39.0 ± 12.3	38.9 ± 12.7	0.937
**BMI (Kg/m^2^)** Normal or thinness Overweight Obese	26.04 ± 5.14 144 (47.5%) 96 (31.7%) 63 (20.8%)	27.90 ± 5.11 45 (31.9%) 57 (40.4%) 39 (27.7%)	24.42 ± 4.59 99 (61.1%) 39 (24.1%) 24 (14.8%)	<0.001 <0.001
**Blood Pressure (BP) (mm Hg)** Systolic Blood Pressure Diastolic Blood Pressure	119 ± 13 76 ± 10	124 ± 13 79 ± 11	115 ± 12 73 ± 9	<0.001 <0.001

Categorical variables are expressed as frequencies and percentages. Continuous variables with Gaussian distribution are expressed as mean ± standard deviation or otherwise as median with its interquartile range (1st quartile–3rd quartile).

**Table 2:** Lipid profile, Lp(a), and PCSK9 in the entire population and in men and women.

	Total Population (n = 303)	Men (n = 141)	Women (n = 162)	*p* Value
TC (mmol/L)	4.9 (4.2–5.60)	4.9 (4.2–5.6)	4.85 ( 4.27–5.5)	0.87
LDL-C (mmol/L)	2.81 (2.10–3.61)	3.12 (2.42–3.83)	2.61 (2.01–3.28)	<0.0001
Non HDL-C (mmol/L) Non HDL-C ≤ 3.3 3.3 < Non HDL-C ≤ 4.1 Non HDL-C > 4.1	3.59 (2.88–3.59) 38.3% 28.7% 33.0%	3.90 (3.19–4.60) 27.7% 28.4% 44.0%	3.39 (2.79–4.05) 47.5% 29.0% 23.5%	<0.0001 <0.0001
Triglycerides (mmol/L) Triglycerides ≤ 1.69 1.69 < Triglycerides ≤ 2.25 2.25 < Triglycerides ≤ 5.65 Triglycerides > 5.65	1.2 (0.85–1.84) 214 70.6%) 34 (11.2%) 50 (16.5%) 5 (1.7%)	1.53 (1.07–2.31) 79 (56%) 20 (14.2%) 38 (27%) 4 (2.8%)	1.02 (0.76–1.48) 135 (83.3%) 14 (8.6%) 12 (7.4%) 1 (0.6%)	<0.0001 <0.0001
HDL-C (mmol/L) <1 mmol/L in men and 1.3 mmoL in women ≥1 mmol/L in men and 1.3 mmoL in women	1.20 (0.98–1.50)	1.01 (0.91–1.2) 44.0% 56.0%	1.45 (1.18–1.66) 37.0% 63.0%	<0.0001 0.22
Lipoprotein a (nmol/L) Lp(a) ≤ 7 7 < Lp(a) ≤ 75 Lp(a) > 75	28 (9–64) 23.1% 57.8% 19.1%	24 (6–51.50) 27.0% 59.6% 13.5%	35.5 (10–69) 19.8% 56.2% 24.1%	0.044 0.044
PCSK9 (ng/mL) PCSK9 ≤ 177 177 < PCSK9 ≤ 460 >460	300 (254–382) 4.3% 80.9% 14.9%	294 (248–364) 4.3% 82.3% 13.5%	308 (259–398) 4.3% 79.6% 16.0%	0.18 0.82

Categorical variables are expressed as frequencies and percentages. Continuous variables are expressed as median with its interquartile range (1st quartile–3rd quartile).

**Table 3:** Correlation matrix of PCSK9 (ng/mL), age (years), BMI (Kg/m^2^), HDL (mmol/L), TG (mmol/L), and non-HDL cholesterol (mmol/L).

		Lpa (nmol/L)	PCSK9 (ng/mL)	Age (Years)	BMI (Kg/m^2^)	HDL (mmol/L)	TG (mmol/L)
**PCSK9** **(ng/mL)**	**CC** **(95%CI)**	0.108 (−0.006; 0.218)					
	***p*-value**	0.062					
**Age** **(years)**	**CC** **(95%CI)**	0.091 (−0.022; 0.203)	0.221 (0.109; 0.327)				
	***p*** **-value**	0.112	<0.001				
**BMI** **(Kg/m^2^)**	**CC** **(95%CI)**	0.034 (−0.079; 0.146)	0.086 (−0.028; 0.197)	0.265 (0.155; 0.369)			
	***p*** **-value**	0.553	0.136	<0.001			
**HDL** **(mmol/L)**	**CC** **(95%CI)**	0.128 (0.015; 0.239)	−0.01 (−0.122; 0.103)	−0.048 (−0.16; 0.065)	−0.400 (−0.494; −0.298)		
	***p*** **-value**	0.025	0.866	0.402	<0.001		
**TG** **(mmol/L)**	**CC** **(95%CI)**	−0.063 (−0.174; 0.051)	0.212 (0.101; 0.319)	0.325 (0.218; 0.425)	0.386 (0.282; 0.48)	−0.593 (−0.665; −0.511)	
	***p*** **-value**	0.277	<0.001	<0.001	<0.001	<0.001	
**Non-HDL** **(mmol/L)**	**CC** **(95%CI)**	0.04 (−0.073; 0.152)	0.221 (0.11; 0.328)	0.391 (0.288; 0.485)	0.297 (0.188; 0.398)	−0.359 (−0.456; −0.254)	0.573 (0.488; 0.647)
	***p*** **-value**	0.489	<0.001	<0.001	<0.001	<0.001	<0.001

**Table 4:** Multiple linear regression analysis with lipoprotein a (Lp(a)) as a dependent variable and gender, age, BMI, SES, non-HDL-C, triglycerides, HDL-C, and PCSK9 as independent variables.

Model		Unstandardized Coefficients		Standardized Coefficients	T	*p*-Value
		B	Std. Error	Beta		
	**(Constant)**	**−0.188**	1.560		−0.120	0.904
	Ln BMI	0.243	0.370	0.043	0.657	0.512
	Age	0.007	0.006	0.083	1.310	0.191
	Gender	0.110	0.151	0.050	0.727	0.468
	Ln (triglycerides)	−0.241	0.160	−0.125	−1.507	0.133
	Ln (HDL-C)	0.305	0.318	0.077	0.957	0.339
	Ln (non-HDL-C)	0.354	0.286	0.088	1.238	0.217
	Ln (PCSK9)	0.320	0.184	0.105	1.740	0.083
a. Dependent Variable: Ln Lp(a)						

Due to their positively skewed distribution, lipid parameters were entered into the model using their natural logarithmic transform. BMI: Body Mass index; Gender (Male = 1; Female = 2). B: unstandardized linear coefficient. Model’s R^2^ = 0.055.

**Table 5:** Multiple linear regression analysis with PCSK9 as a dependent variable and gender, age, BMI, SES, non-HDL-C, triglycerides, and HDL-C as independent variables.

Model		Unstandardized Coefficients		Standardized Coefficients	t	*p*-Value
		B	Std. Error	Beta		
	**(Constant)**	**5.243**	0.385		13.632	0.000
	Ln(BMI)	−0.005	0.117	−0.003	−0.042	0.967
	Age	0.004	0.002	0.124	2.046	0.042
	Gender	0.078	0.048	0.108	1.637	0.103
	Ln (triglycerides)	0.154	0.050	0.242	3.087	0.002
	Ln (HDL-C)	0.125	0.100	0.097	1.248	0.213
	Ln (non-HDL-C)	0.130	0.090	0.099	1.449	0.148
	Ln (Lp(a)	0.032	0.018	0.097	1.740	0.083
a. Dependent Variable: Ln (PCSK9)						

Due to their positively skewed distribution, lipid parameters were entered into the model using their natural logarithmic transform. BMI: Body Mass index; Gender (Male = 1; Female = 2). B: unstandardized linear coefficient. Model’s R^2^ = 0.123.

## Data Availability

Data are available upon request from the corresponding author.
